# Live birth achieved despite the absence of ejaculated spermatozoa and mature oocytes retrieved: a case report

**DOI:** 10.1007/s10815-021-02070-y

**Published:** 2021-01-20

**Authors:** Zuzana Holubcová, Pavel Otevřel, Marek Koudelka, Soňa Kloudová

**Affiliations:** 1 Reprofit International, Clinic of Reproductive Medicine, Hlinky 122, 60300 Brno, Czech Republic; 2 Department of Histology and Embryology, Faculty of Medicine, Masaryk University, Masaryk University Campus – building A1, Kamenice 3, 625 00 Brno, Czech Republic

**Keywords:** IVF add-ons, Oocyte maturity, Polarized light microscopy, Theophylline, Testicular sperm

## Abstract

The most common reason for in vitro fertilization (IVF) cycle cancelation is a lack of quality gametes available for intracytoplasmic sperm injection (ICSI). Here we present the successful fertility treatment of the couple affected by obstructive azoospermia combined with suboptimal response to controlled ovarian stimulation. Since the conventional approach appeared ineffective to overcome both partnersˈ specific problems, the targeted interventions, namely, (1) pharmacological enhancement of sperm motility and (2) polarized light microscopy (PLM)-guided optimization of ICSI time, were applied to rescue the cycle with only immature oocytes and immotile testicular sperm retrieved. The treatment with theophylline aided the selection of viable spermatozoa derived from cryopreserved testicular tissue. When the traditional stimulation protocol failed to produce mature eggs, non-invasive spindle imaging was employed to adjust the sperm injection time to the maturational stage of oocytes extruding a polar body in vitro. The fertilization of 12 late-maturing oocytes yielded 5 zygotes, which all developed into blastocysts. One embryo was transferred into the uterus on day 5 post-fertilization, and another 3 good quality blastocysts were vitrified for later use. The pregnancy resulted in a full-term delivery of a healthy child. This case demonstrates that the individualization beyond the standard IVF protocols should be considered to maximize the chance of poor-prognosis patients to achieve pregnancy with their own gametes.

## Introduction

Assisted reproductive techniques (ART) evolved from simple co-incubation of gametes into a broad repertoire of procedures helping to deal with various fertility issues and avoid transmission of genetic diseases. The concept of controlled ovarian stimulation was developed to provide multiple eggs for insemination by ejaculated sperm. However, patients show variability in their response to hormonal treatment [1, 2]. Occasionally, oocyte-cumulus complexes recovered from preovulatory follicles contain only immature oocytes instead of mature eggs. In the absence of metaphase II (MII) oocytes, the cycle is typically canceled, and immature oocytes are discarded. Failure to complete maturation in the expected timeframe might reflect intrinsic disturbances that compromise female gameteˈs developmental potential [3–6]. Nevertheless, some metaphase I (MI) oocytes which extruded a polar body (PB) in vitro were shown to be clinically utilizable and give rise to pregnancies [7–10]. Ensuring that developmentally delayed oocytes reached the MII stage before ICSI prevents untimely sperm injection and enhances the chance of successful fertilization [11–14]. Polarized light microscopy (PLM) serves as a tool to inspect the presence of MII spindle and time ICSI in accordance with the oocyteˈs maturational stage [15]. Late-maturing oocytes capable of assembling a PLM-detectable MII spindle increase the pool of injectable eggs, thus providing hope for women whose response to gonadotropins is inadequate.

Similarly, testicular sperm extraction (TESE) constitutes the last recourse for men who have no sperm in their ejaculate [16, 17]. However, immature spermatozoa retrieved from extracted testicular tissue are often pathological and immotile. Compounds interfering with phosphodiesterase (PDE) activity, such as pentoxifylline, are known to raise intracellular cyclic adenosine monophosphate (cAMP) and boost motility in asthenospermic samples. Similarly, incubation of TESE-derived sperm with another PDE inhibitor, dimethylxanthine theophylline, enhances sperm movement, thus allowing embryologists to distinguish between viable, yet immotile, and non-viable male gametes [17–19]. So far, no negative effects of sperm stimulation were reported on ICSI outcomes [20–27]. Nevertheless, the potential risks related to the drug exposition still raise safety concerns.

This report provides detailed information on how the application of optional ART techniques, namely, (1) pharmacological stimulation of motility in frozen-thawed testicular sperm and (2) PLM-guided optimization of ICSI time for oocytes extruding PB in vitro, helped to achieve a successful pregnancy under extremely challenging circumstances.

## Results

### Patients’ characteristics and previous treatment

In 2016, a couple with a history of 3 failed attempts performed at a different IVF center approached the clinic presenting with primary male infertility due to the congenital bilateral absence of vas deferens and risk of ovarian hyperstimulation syndrome (OHSS). The previous genetic testing revealed that the 31-year-old male patient was a CTFR gene carrier while his 32-year-old partner was a healthy homozygote. He was referred for TESE, and spermatozoa isolated from the freshly retrieved testicular biopsy were used for ICSI. The rest of the testicular sperm sample was cryopreserved. The nulligravid non-obese female with oligomenorrhea was diagnosed with polycystic ovaries based on ultrasound examination and hormonal profile. To ameliorate the risk of OHSS, she received metformin pre-treatment. Subsequently, the patient underwent ovarian stimulation using antagonist protocol and recombinant follicle-stimulating hormone (rFSH) (Gonal-f 112 IU, Merck Serono, Switzerland). Human chorionic gonadotropin (hCG) (Pregnyl 5.000 IU, Organon, Netherlands) was used to induce ovulation. The oocyte pickup was scheduled for 35 h after the triggering injection. The cumulus-oocyte complexes were retrieved using ultrasound-guided transvaginal aspiration and collected in dual buffered MHM medium (#90166, Irvine Scientific, USA). The oocyte denudation was carried out 10 min after retrieval to distinguish the in vivo matured eggs from oocytes that complete maturation in vitro and benefit from prolonged incubation before ICSI [14, 28]. Despite a good follicular count, no PB-displaying oocytes were obtained. To allow completion of the maturational program, retrieved MI oocytes were placed into CSCM-C medium (#90165, Irvine Scientific, USA) covered with mineral oil and incubated at 37 °C with a humidified atmosphere of 5% O_2_ and 6% CO_2_. Four hours later, 2 out of 7 immature oocytes exhibited a PB and were injected with TESE-derived spermatozoa. Both oocytes showed two well-defined pronuclei 17–18 h after ICSI, but no blastocyst was obtained.

### Case report

In 2018, the couple returned to the clinic to start another cycle. This time, the stimulation phase was prolonged to 13 days of rFSH application (Puregon 125 IU, MSD, USA) to promote follicular growth. The trigger shot was scheduled for 2 days after the biggest follicles reached a diameter of 18 mm, and oocyte retrieval was undertaken 36 h after the hCG injection. Nevertheless, no mature eggs but 17 immature oocytes were retrieved. Only 1 out of 17 MI oocytes displayed a PB after 3 h of in vitro incubation, at standard ICSI time. However, this oocyte did not show an MII spindle signal during the PLM examination, indicating that the maturation process was not yet completed. Therefore, ICSI was postponed, and the maturation stage of each oocyte was closely monitored, as previously described [28]. During the next 2 h, another 4 oocytes extruded PBs. However, they all lacked a PLM-detectable MII spindle signal. Sperm injection was undertaken 2 h later (43 h after hCG) when a total of 12 oocytes exhibited PBs, and 10 of them showed MII spindles. The maturational timelines of individual oocytes, together with their fertilization outcomes, are summarized in Table 1.

**Table 1:** Maturity status of individual oocytes and their post-fertilization outcome

Oocyte #	hCG + 38 h	hCG + 43 h	Fertilization	Blastulation	Utilization
	stage/spindle	stage/spindle			
1	MII/no	MII/yes	No	No	No
2	MI/NA	MII/yes	No	No	No
3	MI/NA	MII/yes	Yes	Yes	Yes
4	MI/NA	MII/yes	Yes	Yes	No
5	MI/NA	MII/yes	No	No	No
6	MI/NA	MII/yes	Yes	Yes	Yes
7	MI/NA	MII/yes	Yes	Yes	Yes
8	MI/NA	MII/yes	No	No	No
9	MI/NA	MII/yes	No	No	No
10	MI/NA	MII/yes	Yes	Yes	Yes
11	MI/NA	MII/no	No	No	No
12	MI/NA	MII/no	No	No	No

The sample derived from the male patient’s testicular biopsy was thawed, but all recovered spermatozoa exhibited pathological morphology and imperceptible spontaneous motility. To prevent ICSI with non-viable spermatozoa, we pretreated the sample with the theophylline-containing medium (SpermMobil, GM501, Gynemed, Germany) for 10 min according to the manufacturerˈs instructions. Short-term exposure to theophylline immediately promoted residual sperm motility and decreased the time necessary to find the best available male gametes for the injection. Each spermatozoon selected for fertilization was extensively rinsed at least three times in the MHM medium before ICSI. Sperm-injected oocytes were transferred to individual droplets of CSCM-C medium, and their post-fertilization fates were tracked.

The next day, 5 out of 12 sperm-injected oocytes showed signs of fertilization. These 5 embryos (all originating from spindled oocytes) reached the blastocyst stage after extended cultivation. Trophectoderm biopsy and genetic testing were proposed, but the couple made an informed decision to proceed with one fresh embryo transfer on day 5 and 3 good quality blastocysts cryopreserved. A singleton pregnancy culminated with spontaneous vaginal delivery at the 38th week of gestation. The male newborn had a normal birthweight (3010 g), and no congenital defects were reported. The child’s well-being was confirmed at the age of 1 year by a detailed survey and will be further monitored to evaluate the treatmentˈs safety.

## Discussion

Over 40 years ago, human IVF emerged as a highly controversial experimental technique. Nowadays, fertility clinics are urged to refrain from advertising unproven methods [29]. The recently developed traffic light system disproved the use of add-on procedures until credible evidence on safety and effectivity is provided [30]. However, many ART techniques routinely used today have been introduced into clinical practice even though their benefits were not demonstrated in randomized controlled trials. Moreover, some fertility issues affect only a small population of IVF patients, and large clinical studies are difficult to design. Innovations are supposed to drive ART advancement, but rigid demand for evidence-based practice might hinder building supportive evidence for novel techniques and alternative approaches [31]. Sharing experience concerning a bona fide use of unconventional interventions and adjuvant therapies is essential in rare patient phenotypes. Clinicians are obliged to monitor the obstetrical and neonatal complications and report any adverse effects that may be recognized. Synthesis and assessment of a “critical mass” of clinical data is a vital prerequisite to evaluate the efficacy of empirical treatments that hold the potential to improve management of ART cycles with bad prognosis.

Here we describe a peculiar case in which the modification of conventional fertility treatment made a difference for the couple with little chance to conceive using traditional IVF protocols. The published data support the use of sperm motility enhancement with theophylline [23–27] and rescue in vitro maturation of late-maturing oocytes [11–14]. Nevertheless, until a sufficient body of evidence is accumulated, these methods must still be regarded as experimental. Our patients were thoroughly counseled over the character of the treatment, and written informed consent was obtained. The follow-up revealed no pregnancy complications or anomalies in the offspring. The health parameters of the child will be further monitored to assess the long-term consequences of the treatment. Based on this experience and previous reports, we suggest considering this approach to salvage cycles with a lack of gametes available for fertilization.

There are some critical aspects and limitations of the procedures in question which need to be acknowledged. Pharmacological stimulation enables faster and more accurate identification of viable sperm in pathological samples, thus increasing the chance for a successful outcome in IVF cycles affected by severe male infertility factor. Availability of ready-to-use and quality-controlled theophylline-containing commercial media makes the application of sperm motility enhancement possible in countries where self-preparing and in-house supplementation of IVF-used solutions are prohibited. Brief exposition to motility-inducing agents appears not to impair sperm genetic integrity. Nevertheless, extensive washing of selected spermatozoa is highly recommended as a precaution to prevent adverse effects on oocytes and embryos [17, 32]. Since spermatogenic impairment was found to increase the risk of embryonic mosaicism, preimplantation genetic testing of ICSI outcomes is advisable when TESE-derived sperm is used [33–35].

The principle and methodology of PLM examination, as well as the optimization of the ICSI timing, have been thoroughly described previously [14, 15, 28, 36]. However, it is important to emphasize here that spindle imaging provides no information about chromatin configuration at the metaphase plate. Chromosome misalignment and precocious chromatid splitting could occur in oocytes featuring the MII spindle [28, 37]. Therefore, younger patients with an unexpectedly inadequate response to ovarian stimulation due to specific genetic background and/or endocrine derangements [38–40] are more likely to benefit from PLM-navigated ICSI timing than women of advanced age.

In conclusion, our patient’s story highlights the need for personalization and responsible innovation in reproductive medicine. While unproven therapies cannot be recommended in a general context, the judicious use of specific unconventional interventions is justifiable as an alternative to cycle cancelation.
